# Transovarial Transmission of Cell-Fusing Agent Virus in Naturally Infected *Aedes aegypti* Mosquitoes

**DOI:** 10.3390/v16071116

**Published:** 2024-07-11

**Authors:** Dilip K. Nag, Kathryn J. Efner

**Affiliations:** Griffin Laboratory, Wadsworth Center, New York State Department of Health, Slingerlands, NY 12159, USA; kjefner@gmail.com

**Keywords:** arbovirus, insect-specific virus, orthoflavivirus, cell-fusing agent virus, mosquitoes, vertical transmission

## Abstract

Mosquito-borne arboviruses include several pathogens that are responsible for many diseases of significant public health burden. Mosquitoes also host many insect-specific viruses that cannot replicate in vertebrate cells. These insect-specific viruses persist in nature predominantly via vertical transmission (VT), and they exhibit high VT rates (VTRs). Cell-fusing agent virus (CFAV), an insect-specific orthoflavivirus, shows high VTRs in naturally infected mosquitoes but not in artificially infected mosquitoes. To determine whether the high VTRs are due to transovarial transmission, we investigated VT and ovary infection patterns in naturally CFAV-infected *Aedes aegypti* (Bangkok) mosquitoes. VT was monitored by detecting CFAV among the progeny by reverse-transcription polymerase chain reaction and ovary infection was determined by in situ hybridization using a virus-specific probe. We showed that in CFAV-positive mosquitoes, ovarian follicles were infected, suggesting that VT occurs by transovarial transmission in naturally infected mosquitoes. Additionally, mosquitoes harbored dormant, non-replicative CFAV that remained below the detection level. These results suggested that CFAV persists via VT in nature and has the potential to remain dormant in diapausing mosquitoes during unfavorable conditions. Understanding this VT mechanism is crucial for comprehending the persistence of insect-specific viruses (and potentially dual-host arboviruses) in their natural environment.

## 1. Introduction

Mosquitoes are the major vectors of several medically important arthropod-borne viruses (arboviruses), including West Nile virus (WNV), dengue virus (DENV), Zika virus (ZIKV), and Chikungunya virus (CHIKV) [1]. These viruses are maintained in nature in cycles involving susceptible vertebrate hosts and mosquito vectors. During permissive conditions, arboviruses are maintained via horizontal transmission between arthropod vectors and vertebrate hosts, allowing amplification of the virus in nature. However, the mechanisms by which these viruses are maintained during unfavorable environmental conditions, such as during dry seasons in tropical areas and cold seasons in temperate regions, are poorly understood. These conditions may lead to low vector population or diapausing vectors, interrupting the transmission cycle. Vertical transmission (VT), the transmission of pathogens from infected parents to their offspring, can provide an essential link in the transmission cycle during adverse environmental conditions. In addition, VT can maintain a virus in a region where the number of susceptible vertebrate hosts is low due to vaccination or herd immunity. 

**Figure 1:**
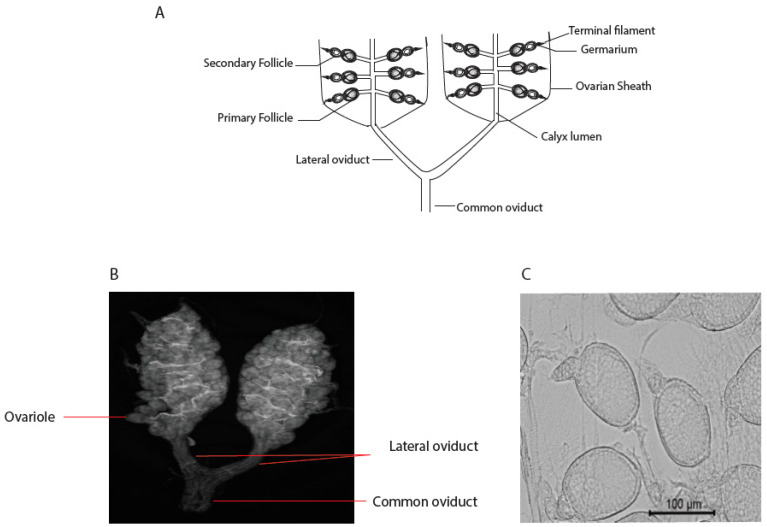
(**A**) A schematic diagram showing part of the mosquito reproductive system (after ref. [3]). (**B**) An image of *Aedes aegypti* ovaries. (**C**) Picture of *Ae. aegypti* ovarioles.

For efficient VT to occur, the virus must infect the mosquito reproductive tissue. The reproductive system of female mosquitoes consists of two ovaries located in the area of the fifth abdominal segment; they connect with a pair of lateral oviducts that join to form a common oviduct opening posteriorly into a genital chamber. Each ovary is made of 50–100 ovarioles, where the eggs are produced. Each ovariole contains a group of germ cells (oogonia) that divide by mitosis and produce oocytes near the distal end, followed by two follicles and a pedicel attaching them to a central canal, the calyx, which ultimately becomes the lateral oviduct (Figure 1). Each follicle consists of follicular epithelium, nurse cells, and oocytes. A majority of egg-shell structural components are secreted by the follicular epithelial cells [2]. After maturation of the primary follicle and release of the egg, the secondary follicle becomes the primary follicle, and the cycle continues. 

VT of arboviruses by female mosquitoes can occur via two pathways: transovarial transmission (TOT), where the arbovirus infects the ovarian follicles, leading to infection of the mosquito progeny, or transovum transmission, where the infection occurs at the time of fertilization via the micropyle or due to contamination of the eggs during oviposition [4]. Once the virus establishes infection of the ovarian follicles and germariums, a high proportion of progeny are infected transovarially in successive generations (defined as stabilized infection). VT varies between virus families and even among viruses within the same genus [4,5,6,7,8,9,10,11,12,13,14,15,16,17]. For example, orthobunyaviruses (e.g., La Crosse virus [LACV]) exhibit high filial infection rates (FIRs), whereas orthoflaviviruses (e.g., DENV and ZIKV) and alphaviruses (e.g., CHIKV) have inefficient VT and are believed to be transmitted vertically via transovum transmission, as FIRs have been found to be quite low. 

Efficient VT protects the virus in diapausing eggs during adverse environmental conditions and can also amplify the number of infected mosquitoes in successive gonotrophic cycles (the cycle of blood feeding, egg development, and egg laying) during permissive conditions. Even for viruses that exhibit high FIRs, most models predict that significant horizontal transmission is necessary for the maintenance of these viruses in nature. For example, LACV survives the winter in the diapausing eggs of its mosquito vector *Aedes triseriatus,* and TOT is essential for LACV overwintering and amplification in nature [4,5,18]. However, FIRs observed in the laboratory appear to be insufficient to maintain the virus in nature. These observations suggest that both horizontal and VT are necessary to sustain arboviruses in nature. 

There is a growing list of viruses, known as insect-specific viruses (ISVs), that do not replicate in vertebrate cells, and instead grow only in insect cells [19]. Although they are called insect-specific, most of them were discovered in mosquitoes. Since there are no amplifying vertebrate hosts, these viruses are maintained in nature via VT and venereal transmission, as well as potentially through horizontal transmission during larval stages in the vector’s aquatic environment [20,21,22,23,24]. Cell-fusing agent virus (CFAV), an orthoflavivirus, was the first ISV isolated from an *Aedes aegypti* cell line [25]. Since then, a large number of ISVs have been isolated due to advancement in high-throughput sequencing, metagenomics, and enhanced mosquito surveillance. These ISVs show a high rate of VT in naturally infected mosquitoes. Gaining insight into the mechanisms of VT that support the persistence of ISVs in nature may shed light on how dual-host (mosquito and vertebrates) arboviruses are maintained in natural environments. 

Among the ISVs, insect-specific orthoflaviviruses (ISOFs) are relatively well studied. ISOFs recently received significant attention because of their potential to modulate infection and transmission of pathogenic arboviruses [26,27,28,29,30,31,32,33,34,35,36]. While ISVs show a high rate of VT in field-collected mosquitoes, VT in artificially infected, laboratory-colonized mosquitoes is rare to non-existent, although viral RNA can be detected in mosquito ovaries [37,38,39,40]. For example, CFAV, *Culex* flavivirus, and Eilat virus (an insect-specific alphavirus) rarely transmit vertically in laboratory-colonized mosquitoes. In situ hybridization (ISH) has also revealed the presence of CFAV in mosquito ovaries in intrathoracically infected mosquitoes, but no ovarioles were infected. Here, we sought to understand how CFAV is vertically transmitted in naturally infected mosquitoes and whether TOT is responsible for the observed high FIRs in naturally infected mosquitoes. Our results indicated that VT of CFAV in field-collected mosquitoes occurs via TOT. 

## 2. Materials and Methods

**Mosquitoes**. Mexican *Ae. aegypti*, graciously supplied by GD Ebel of Colorado State University, were initially gathered in Poza Rica, Mexico. Bangkok *Ae. aegypti* were sourced from Bangkok and then reared in the laboratory. These mosquitoes were generously provided by Nikos Vasilakis of the University of Texas medical Branch in Galveston, Texas. The mosquitoes were bred in the insectary and kept at 28°C with a 16/8-h light/dark cycle. Larvae were nourished with ground fish food, and the adults were maintained on a 10% sucrose solution. The F35 generation of Mexican and F1 generation of Bangkok *Ae. aegypti* were used for our experiments. The generation number of Bangkok mosquitoes was unknown when we received them from the Vasilakis laboratory. 

**Virus.** CFAV ([Galveston], NC001564) was obtained from the World Reference Center for Emerging Viruses and Arboviruses, University of Texas Medical Branch, Galveston, TX, USA. CFAV was isolated from a pool of 50 females (Galveston Colony), passaged twice in C6/36 cells before we received them and then once in C6/36 cells (ATCC, CRL-1660) to generate our stock virus. C6/36 cells were grown in MEM supplemented with 10% FBS, 1.5 g/L sodium bicarbonate, 0.1 mM non-essential amino acids and were maintained at 28°C in 5% CO_2_. All media had 100 U penicillin mL^−1^ and 100 μg streptomycin mL^−1^. Confluent monolayers of C6/36 cells in six-well plates were infected with CFAV at 0.1 multiplicity of infection (MOI). After one hour of adsorption, 3 mL of maintenance medium (growth medium with 2% FBS) was added to each well, and the plates were incubated at 28°C. Supernatants from each well were collected after 7 days and pooled. 

**Determination of VT in *Ae. aegypti* (Bangkok) mosquitoes.** To determine VT, mosquitoes from the colony were fed with a non-infectious blood meal after being starved for 24 h. Engorged mosquitoes were pooled in a cardboard container and maintained with 10% sucrose at 28°C. After two days, mosquitoes were separated into individual cups containing an oviposition substrate [40]. On day 5 post feeding (after the eggs were laid), the midguts and ovaries were dissected and fixed with 10% neutral-buffered formalin for 2–7 days at 4°C to determine the tissue infection patterns by CFAV. Carcasses were collected in 500 μL mosquito diluent (1×PBS containing 20% heat-inactivated FBS, 100 U/mL of Penicillin, 100 μg/mL Streptomycin, 10 μg/mL Gentamycin, and 1 μg/mL of Fungizone) containing a 4 mm bead (Daisy Rogers, Rogers, AR, USA), to determine the infection status of mosquitoes. Samples were kept at −80°C prior to RNA isolation. The infection status was determined by a reverse transcriptase quantitative polymerase chain reaction (RT-qPCR) assay. 

RNA was extracted from homogenized samples using MagMax nucleic acid isolation chemistry (Applied Biosystems, Waltham, MA, USA). All real-time assays were performed by using the qScript XLT 1-step RT-qPCR kit (Quantabio, Beverly, MA, USA) with amplification in the QuantStudio 5 instrument (Applied Biosystems) following the manufacturer’s protocol. C_T_ values of 36 and over were not considered as positive, as they produced inconsistent results. F1 progeny were fed again with a non-infectious blood meal and maintained in individual cups with oviposition substrates. After the eggs were laid, mosquitoes were dissected as above, and the infection status of the mosquitoes and tissue infection patterns were determined by RT-qPCR and ISH, respectively. F2 mosquitoes were raised to the adult stage and the infection status was determined as described above. ISH was performed as described previously [40]. 

VT rate (VTR) is defined as the number of infected females in a population that produce at least one infected offspring. The FIR is the total number of positive progeny divided by the total number of progeny. CFAV primers are as follows: Forward, 5′-CCATTGCGACAGAGGATTCA-3′; Reverse, 5′-GTGTCGCTAACAGAGTGGAAG-3′; Probe, 5′-/5Cy5/TTCCATCGC/TAO/TAGGTCAGCCATTGT/3IAbRQSp/-3′. The primers were synthesized at Integrated DNA Technologies, Coralville, IA, USA. 

## 3. Results

**Figure 2:**
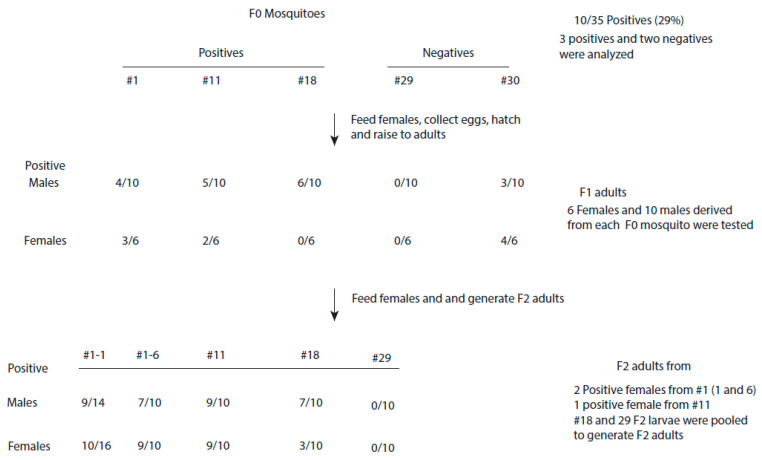
Diagram indicating vertical transmission of cell-fusing agent virus in two generations of *Aedes aegypti* (Bangkok) mosquitoes. Also shown are the infection rates in three generations of mosquitoes.

**VT of CFAV in naturally infected mosquitoes**. To monitor VT of CFAV, *Ae. aegypti* (Bangkok) mosquitoes from the colony were fed with a non-infectious blood meal. Engorged mosquitoes were separated into individual cups containing oviposition substrates. After five days, the eggs were collected, and the mosquitoes were dissected to determine the infection patterns of the midguts and ovaries (see Section 2). The carcasses were used to determine the infection status of the mosquitoes by RT-qPCR using virus-specific primers and probes. A total of 36 female mosquitoes were used (Figure 2). One mosquito died prematurely. Ten out of the remaining thirty-five mosquitoes (29%) were infected with CFAV, with C_T_ values ranging from 30 to 32. From these 35 mosquitoes, 5 random mosquitoes (3 positives and 2 negatives) were used for further analysis. Adult F1 mosquitoes derived from each F0 mosquito were pooled and then fed with a non-infectious blood meal. From the pool, 10 males were tested for their infection status by RT-qPCR, and 6 engorged females were separated into individual cups containing oviposition substrates. After the mosquitoes had laid their eggs, they were dissected. The ovaries and midguts were fixed with formalin, and the carcasses were collected in mosquito diluent to determine the infection status of the mosquito (Figure 2). The carcasses were triturated and suspended in diluent for testing to determine the infection status. 

All three F0 positives produced infected progeny, resulting in a 100% VTR. Forty to sixty percent of F1 males were infected with CFAV (Figure 2). However, only two F0 females (#1 and 11) produced infected F1 females. None of the six females produced from #18 F0 mosquito were CFAV-positive by the RT-qPCR assay. From the two F0 negative mosquitoes, one mosquito (#30) produced infected progeny, while none of the progeny derived from #29 were infected with the virus (Figure 2). Thirty percent of males and sixty-seven percent of females produced by the #30 mosquito were infected with the virus. These results suggested that the #30 mosquito was infected with CFAV and must have had a virus concentration below the detection level of the RT-qPCR assay. The virus likely remained in this mosquito in a non-replicating dormant state. 

Two F1 CFAV-positive females from #1 and one from #11 were given another non-infectious blood meal to generate F2 adults. They were kept separately in individual cups. Six F1 female mosquitoes each from #18 and #29 were fed another blood meal. Since these mosquitoes were CFAV-negative, the larvae derived from #18 and from #29 were pooled separately and raised to the adult stage. Ten to sixteen F2 adult males or females were tested by RT-qPCR to determine their infection status. Both females from #1 produced infected progeny. Sixty-three to ninety percent of the progeny were infected (Figure 2). None of the F2 adults derived from #29 F1 mosquitoes were infected with the virus. Among the F2 progeny derived from the #18 F1 mosquitoes, 70% of males and 30% of females were infected. These results again suggested that #18 F1 females, although negative by RT-qPCR assay, were infected with the virus, which remained in the dormant undetectable state. We repeated this experiment with another 20 mosquitoes and obtained similar results, where an uninfected F1 female derived from an infected F0 female produced infected F2 progeny. 

**Transovarial transmission of CFAV**. In previous studies with artificially infected laboratory-colonized mosquitoes, VT of CFAV was found to be rare [39,40]. Although the ovaries were infected, the ovarian follicles were not infected [40]. Since the naturally infected *Ae. aegypti* (Bangkok) mosquitoes exhibited high FIRs, we wished to determine the ovary infection patterns in these mosquitoes to ascertain whether VT in *Ae. aegypti* (Bangkok) mosquitoes occurs via TOT or transovum transmission. The ovaries were dissected and fixed with formalin, and the ovary infection pattern was determined by ISH, using a virus-specific probe, as described previously [40]. Since we did not have *Ae. aegypti* (Bangkok) mosquitoes that had not been exposed to CFAV, we used Mexican *Ae. aegypti* as a control, which have previously been shown to be CFAV-negative [40]. 

**Figure 3:**
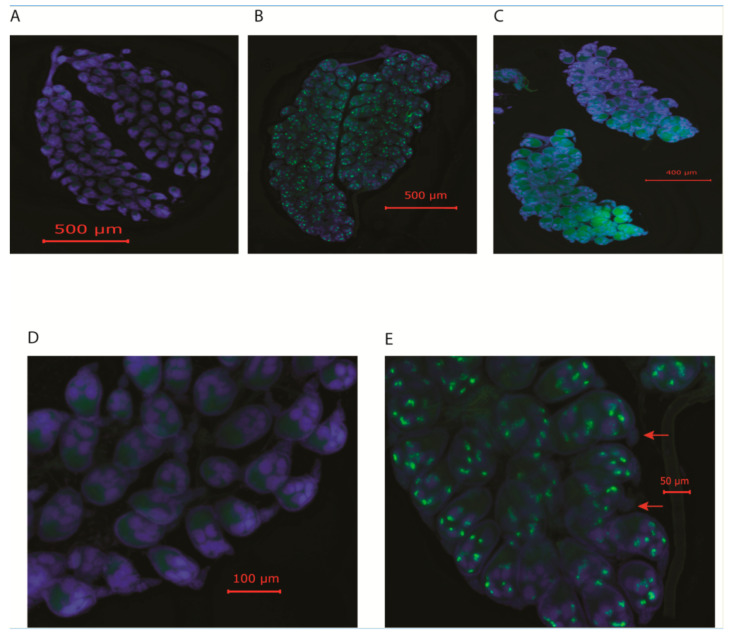
Cell-fusing agent virus (CFAV) infects ovarian follicles in naturally infected *Aedes aegypti* (Bangkok) mosquitoes. Mosquitoes were dissected 5 days post blood meal and after the mosquitoes had laid eggs. Ovaries were fixed with 10% formalin, and viral presence was determined by in situ hybridization. *Ae. aegypti* (Mexico) were used as control. (**A**,**D**), *Ae. aegypti* (MX) uninfected ovaries at 10× and 40× magnifications, respectively; (**B**,**C**), ovaries from F0 and F1 CFAV-positive mosquitoes, respectively. (**E**), CFAV-positive mosquito ovary at 40× magnifications. Arrows indicate that both germariums and secondary follicles are infected.

To determine the ovary infection pattern, we pooled all the ovaries collected from CFAV-positive F0 and F1 females separately, after they had laid eggs. A total of five ovary pairs were tested. Both ovarian follicles and germariums were infected (Figure 3B,C,E). Most of the fluorescence was associated with the nurse cells. No fluorescence was observed surrounding the follicle (i.e., on the follicular epithelium). No signal was present in the oviducts in any of the ovaries tested. These results suggested that VT of CFAV in naturally infected mosquitoes occurs via TOT. However, infections of the ovarian follicles do not guarantee that all progeny will have a detectable level of the virus. 

The infection patterns of the midguts from CFAV-positive mosquitoes were also tested by ISH. Surprisingly, in the midguts, signals were not detectable (Supplementary Figure S1); midguts from infected and uninfected mosquitoes were indistinguishable. It is possible that only ovaries remain infected in these mosquitoes. To test this possibility, another fifty-two mosquitoes were dissected, and the midguts, ovaries, and carcasses were tested for virus by RT-qPCR. Seven out of fifty-two mosquitoes (14%) were infected with the virus. Our results showed that whenever the ovaries were infected, the midguts and carcasses were also infected, suggesting that although the ovaries exhibited the strongest signals in ISH analyses, the virus was also present in the midgut and carcass. Perhaps, midgut infections are maintained in a manner that cannot be detected by ISH, or the inability to detect the virus in midguts is due to the inefficient target retrieval step of the ISH assay. 

## 4. Discussion

One long-standing puzzling question for arbovirologists is how arboviruses persist in nature during unfavorable environmental conditions, when adult vectors, such as mosquitoes, are absent or present in very low numbers. VT of arboviruses is one mechanism which can provide the missing link in the viral transmission cycle. Viruses can be maintained in diapausing eggs during harsh environmental conditions and a new transmission cycle can be initiated during favorable conditions by the eggs’ eventual production of infected adult mosquitoes. While some viruses, such as orthobunyaviruses, exhibit efficient VT, others like orthoflaviviruses and alphaviruses are poorly transmitted from parent vectors to their offspring, and they are likely transmitted vertically during oviposition when the egg travels through the infected calyx and oviducts. ISVs, on the other hand, show higher VTRs than several dual-host arboviruses [4,19,41], and because of their high VTRs, ISVs offer an excellent system to study the mechanism of VT of arboviruses in mosquito vectors.

There is little doubt that orthobunyaviruses are transmitted vertically via TOT, as viral antigens have been detected within the ovarian follicles [4,42,43]. High VTRs by ISOFs in field-collected mosquitoes are also believed to be due to TOT, where the virus infects the ovarian follicles and produces a large number of infected progeny. However, until now, there has been no physical evidence of infection of ovarian follicles by ISOFs. Here, we demonstrated that CFAV is transovarially transmitted in naturally infected mosquitoes. 

Previously, we reported that a fraction of mosquitoes (30%) in the Bangkok population were infected with CFAV [40]. Similar results were also obtained with another CFAV-infected *Ae. aegypti* population from Thailand [44]. Here, we showed that, on average, 20% (17/87) of female mosquitoes in the population were infected with CFAV. However, this figure is an underestimate, because several RT-qPCR-negative female mosquitoes were, in fact, infected, as they produced infected progeny upon blood feeding. CFAV in these mosquitoes likely remained in a non-replicating dormant state and was maintained at an undetectable level. It appears that some physiological changes associated with blood feeding stimulated viral replication, allowing virus transmission to the progeny. However, blood feeding alone cannot induce non-replicating dormant viruses in all mosquitoes. CFAV-positive F0 females produced an average of 42% CFAV-positive progeny (F1), and the filial infection rate in the F2 generation was increased to 76% (*p* = 0.0002) (Figure 2). Contreras-Gutierrez et. al. [41] obtained both an F1 filial infection rate of 40–100% and an F2 filial infection rate of 100% with a naturally infected Galveston *Ae. aegypti* colony, suggesting that VT may depend on the mosquito population. In addition, the increase in F2 filial infection in the Thailand population may be due to the fact that F1 mosquitoes with dormant viruses produced infectious progeny. 

Infection patterns of the reproductive tissues were also analyzed by ISH using a virus-specific probe. Ovaries derived from CFAV-positive mosquitoes were analyzed. Both primary and secondary follicles, as well as germariums were infected, indicating TOT of CFAV in naturally infected mosquitoes. Most of the fluorescence was associated with the nurse cells. It is unclear at this stage how ovarioles were infected. In artificially infected mosquitoes, CFAV signals were present in the oviducts as for other dual-host orthoflavivirues [15,40,45,46,47], but no CFAV signal was present in the oviducts or follicular epithelium in naturally infected mosquitoes (Figure 3). Either the oviducts are not involved during infection of the ovarioles, or the virus is cleared from the oviducts once the germariums are infected. Infection of tracheal cells is the likely precursor to the ovariole infection. More ovaries need to be tested in order to identify the precursor step with certainty. Other investigators also suggested tracheal cells as a possible conduit for the entry of rift valley fever virus and La Crosse virus into the ovaries [42,48,49]. 

Since these mosquitoes have been naturally infected over a long period of time, it is likely that they also possess DNA forms of the RNA genome, known as endogenous viral elements (EVEs) [50,51,52]. These EVEs control viral replication by producing P-element-induced wimpy testis-interacting RNAs (piRNAs) [44]. The piRNA-mediated antiviral activity is strongest in the ovaries [44], and thus may regulate VT of CFAV. Previous studies have shown that the piRNA mechanism reduces the virus replication in the mosquito vector [44], but does not completely turn off the virus replication. It is unclear at this stage whether piRNA alone is responsible for maintaining CFAV at an undetectable level or if some other unidentified mechanism is also at play. It is also possible that, in mosquitoes where the virus remains at an undetectable level, CFAV might be restricted to pockets of cells rather than infecting a large number of cells, thus maintaining the virus at a low level. These mosquitoes with undetectable levels of the virus produce infected progeny as the virus replicates and spreads throughout the tissue following blood feeding during egg development. Additional studies are necessary to understand how mosquitoes maintain the virus in a non-replicative dormant state. We also studied the infection patterns of the midguts in infected mosquitoes. No difference was observed between the midguts from uninfected (RT-qPCR-negative) and infected (RT-qPCR-positive) mosquitoes (Supplementary Figure S1), suggesting midgut infections persist in a way that is undetectable by ISH. It is also possible that midgut infection could not be detected due to a technical issue. For example, the target retrieval step for the midgut tissue may not have been long enough to be detectable by the virus-specific probe. 

Once the germariums and follicles are infected, stabilized infections are established, which leads to a high level of infected progeny in subsequent generations. Further investigations are necessary to understand how stabilized infections are established. It is also not clear why laboratory infected mosquitoes and field-collected mosquitoes exhibit different ovary infection patterns and VTRs. It is possible, as suggested previously, that genetic differences between the mosquito populations are responsible for different outcomes in the two populations (laboratory- and field-collected) [53,54]. Further studies are needed to clearly understand VT of arboviruses and their maintenance in nature. 

In summary, our results showed that VT of CFAV in naturally infected mosquitoes occurs via TOT. Both follicles and germariums were infected with the virus. We also demonstrated that several females in the population maintained CFAV in a dormant non-replicative state. Some physiological changes associated with blood feeding likely activated virus replication, allowing the virus to be transmitted to the progeny. 

## Data Availability

All data generated or analyzed during this study are included within this article.

## Supplementary Materials

The following supporting information can be downloaded at: https://www.mdpi.com/article/10.3390/v16071116/s1, Figure S1: In situ hybridization analysis of midguts from CFAV-positive and -negative mosquitoes. After dissection, midguts were fixed with 10% formalin and analyzed by in situ hybridization. A, midgut from an uninfected mosquito; B, midgut of an infected mosquito.
