# Supplementary figures and images for: Transovarial Transmission of Cell-Fusing Agent Virus in Naturally Infected Aedes aegypti Mosquitoes
Source: Viruses. 2024 Jul 11;16(7):1116. doi: 10.3390/v16071116 (PMC11281400; doi:10.3390/v16071116)

Supplementary Fig. 1

A

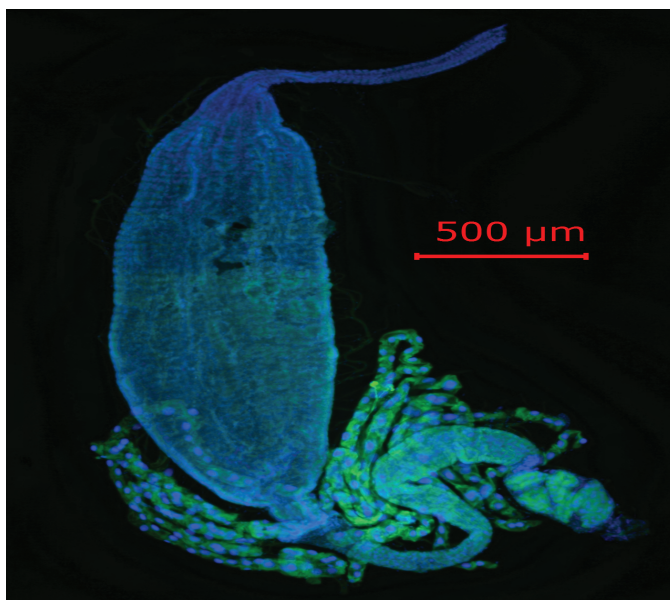

B

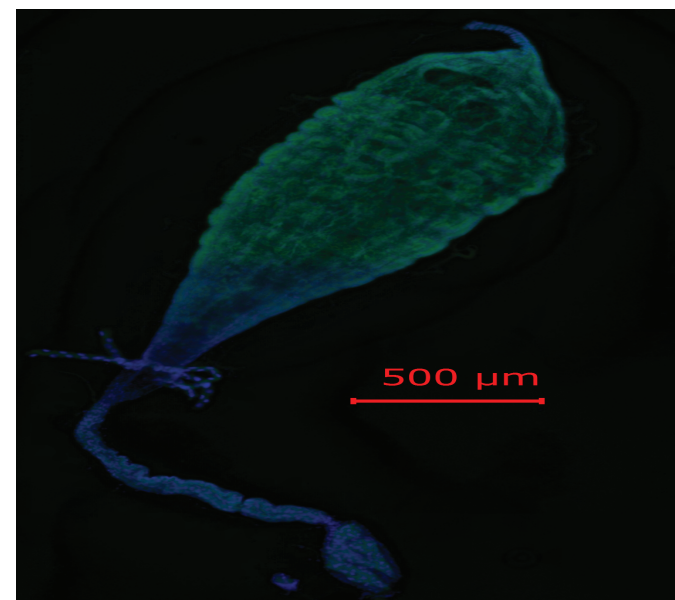

Supplement: Supplementary file 1 [file viruses-16-01116-s001.zip › viruses-3056405-supplementary.pdf]
